# Primary Asthenic Gout by Augustin-Jacob Landre-Beauvais in 1800: Is this the first description of Rheumatoid Arthritis?

**DOI:** 10.31138/mjr.28.4.223

**Published:** 2017-12-22

**Authors:** Gregory Tsoucalas, Markos Sgantzos

**Affiliations:** 1 Department of History of Medicine,; 2 Anatomy Department, Faculty of Medicine, University of Thessaly, Larissa, Greece

**Keywords:** rheumatoid arthritis, gout, Augustin-Jacob Landre-Beauvais, history of rheumatology

## Abstract

Although Alfred Baring Garrod was the physician who introduced the term Rheumatoid Arthritis, it was the French medical student Augustin-Jacob Landre-Beauvais who firstly described the newly appeared type of arthritis. In his opinion, “Goutte Asthénique Primitive”, as he named it, affected mostly the poor, with bigger prevalence among women, presenting characteristic capsular swelling, limitation of motion of the joints in the hands and fingers, and development of bony ankylosis with disorganisation of many joints. Landre-Beauvais was known for his empathy towards his patients, an acquired feeling due to his internship under Pinel. His greatest treatise “Séméiotique, ou Traité des signes des maladies” was greatly influenced in its structure by the concept of Corpus Hippocraticum.

## INTRODUCTION

**Figure 1:**
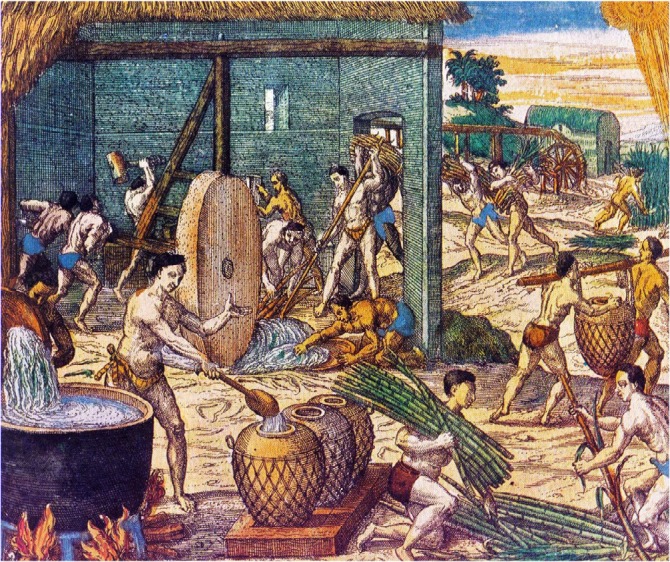
Caribbean sugar cane mill, coloured engraving by Theodor de Bry (1528–1598).

In the eve of the 19^th^ century, rheumatoid arthritis (RA) was introduced as a newly appearing disease among the general European population. A series of hypotheses concerning its prevalence were made, but most probably, sugar was to be blamed.^1–3^ During that era, sugar trade between America and Europe was at its peak. The West Indian sugar trade flourished between 1755–1765 AD (**Figure 1**). Understanding sugar’s commercial significance, the British government taxed molasses and sugar, so that sugar soon became “the white gold of the upper class”. Taxation provoked colonial reaction in 1773, protesting being heavily taxed without representation by dumping tea and sugar into Boston Harbour. By 1771, this tax netted England with 326,000 pounds, while by 1800, sugar consumption was about 160 million lbs per year, providing more than 3 million pounds of revenue by 1815. Tea flavoured with sugar had evidentially reached the middle class. Some decades later, during 1874, British government abolished the tax in order to make sugar accessible to all ordinary British people. It was during that time that tooth decay and periodontal disease increased, and RA appeared.^2,4^

Rheumatoid arthritis is a severe, painful and crippling disease affecting millions of people throughout the world, especially women. Today the evidence for the association between sugar intake, dental decay, chronic periodontitis and incidence of chronic systemic diseases like cancer, atherosclerosis and, in our case, arthritis, is a rather significant probability. While sugar is implicated in the appearance of RA, in his part, RA in a physiology’s circle was implicated in the decline in salivary glandular function associated with a delay in the oral sugar clearance, provoking further aggravation of the RA disease itself.^5–7^

Although the term “rheumatoid arthritis” was introduced by English physician Alfred Baring Garrod (1819–1907) in 1859, it was French medical student, Augustin-Jacob Landre-Beauvais (1772–1840), who in his doctoral thesis “Goutte Asthénique Primitive”, described the first series of patients with classic RA during 1800. ^8,9^

## AUGUSTIN-JACOB LANDRE-BEAUVAIS, HIS LIFE AND WORK

**Figure 2:**
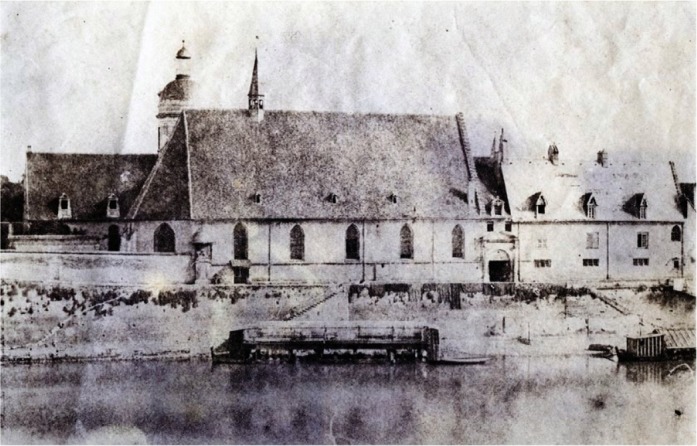
Hospital Chalon-sur-Saône, photo by Joseph Fortune Petiot-Groffier (1788–1855) in 1853.

Augustin Jacob Landré-Beauvais, born in Orléans on April the 4^th^ 1772 (or 1752 as Hippolyte Peisse noted in his treatise “Les médecins Français contemporains” in 1827), was a French surgeon, pupil of the anatomist-surgeon and forerunner of modern medical teaching Pierre Joseph Desault (1738–1795) and anatomist-pathologist and father of histology Marie François Xavier Bi-chat (1771–1802) in Medical School of Paris. Even though his greatest achievement, the first description of RA, was accomplished during his very young age, he remained neglected and very little is known about his life. From 1792, he had been specialized under anato-mist-surgeon and inventor of the screw-type tourniquet Jean Louis Petit (1674–1750) in Lyon. Over the next two years of 1793–1794 he had exercised surgery in the civilian-military Hospital Chalons sur Saône (**Figure 2**). In 1796, he obtained an internship at the famed Salpêtrière Hospital as a physician’s assistant and developer of a more humane psychological approach to the custody and care of psychiatric patients Philippe Pinel (1745–1826). He was appointed Professor of clinical medicine at the Salpêtrière in 1799. In 1814, he became a member of the Légion d’ Honneur. Some years later, in 1825, he was appointed in the Parisian Polytechnic School and became consultant of the King. He was removed in 1830 at the insistence of King Louis Philippe of France (1773–1850).^10–11^

**Figure 3:**
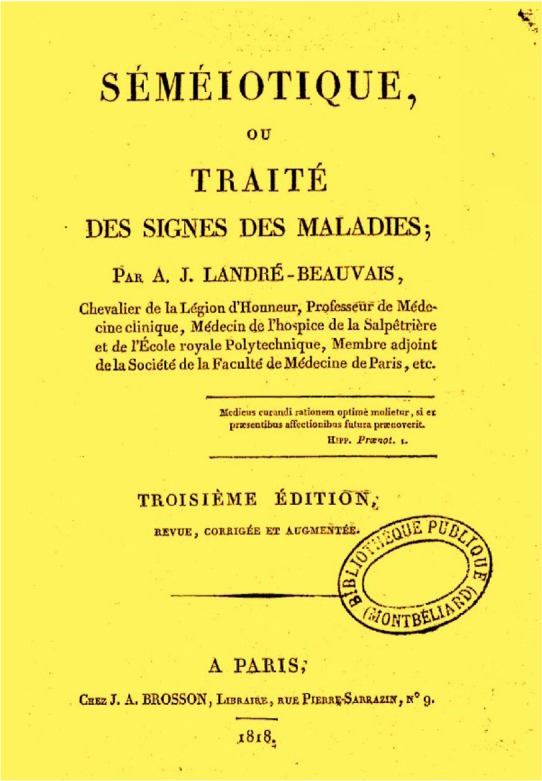
Séméiotique ou Traité des signes by Landré Beauvais Augustin Jacob in 1818.

His extensive education influenced him in a way that is clearly presented in his most significant publication “Séméiotique, ou Traité des signes des maladies” (Semiotics, or Diseases’ signs) (**Figure 3**). In his great catalogue of signs written in 1809, he thoroughly presented the symptoms of a series of diseases similarly to the Hippocratic structure of Corpus Hippocraticum (5th century BC), based upon thorough observation. He had furthermore taken it for granted that anxiety will be present in infectious illnesses, influenced by Pinel’s views and empathy towards patients.^11,12^

## RHEUMATOID ARTHRITIS’ FIRST DESCRIPTION

**Figure 4:**
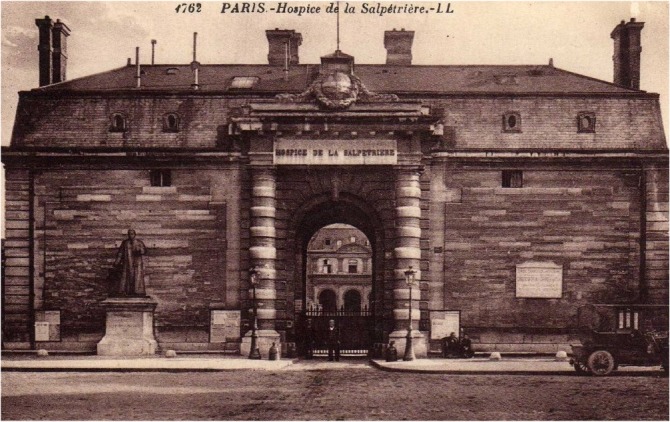
Salpêtrière hospice in Paris, postcard in 1762.

Many physicians in the western European modern medicine, like the French physician Guillaume de Baillou (Latinized: Ballonius) (1538–1616), who recognized rheumatism as being arthritis in 1611, were involved into the study of RA. However, Augustin Jacob Landré-Beauvais was the first to describe it clearly.^13^ His unabridged version of the dissertation presented in 1800 described a disease, different in many ways from the condition known since Hippocrates (ca 460–370 BC) as gout. Landré-Beau-vais was only 28 years old and only a resident physician at the Saltpêtrière asylum in France when he had first noticed the symptoms and signs of what we now know to be RA. He had examined and treated a handful of patients with severe joint pain that could not be explained by other known nosological entities at the time, neither rheumatism, nor osteoarthritis. Unlike gout (podagra, Arthritis urica), which was the disease of the upper class, this condition mainly affected the poor. Furthermore, it affected female patients more often than male, and had previously been ignored by other physicians who usually chose to treat more affluent patients to earn fame and compensation for their work. Landré-Beauvais described a group of nine female long-term residents of the Salpêtrière hospice in Paris (**Figure 4**) with a chronic new type of arthritis. He hypothesized that these patients were suffering from a previously uncharacterized condition, which he named “Goutte Asthénique Primitive” (Primary Asthenic-Debilitating Gout).^14,15^

**Figure 5:**
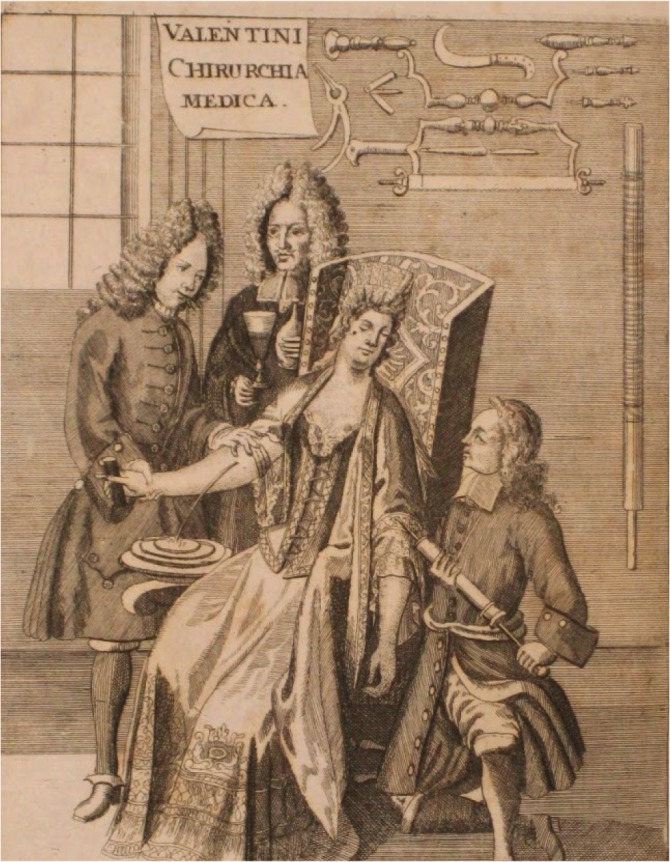
Bloodletting in a female patient, engraving 18^th^ century.

In his opinion, this type of arthritis exhibited several distinctive features; including predominance in women, a chronic course, involvement of many joints from the onset, and a decline in general health, noting “we must recognize the existence of a new form of gout, under the designation ‘primary asthenic gout’”. Landré-Beauvais described a new cluster of symptoms with characteristic capsular swelling, limitation of motion of the joints in the hands and fingers, a condition that may spread to other joints, and eventually development of bony ankylosis with disorganisation of many joints. He had focused in a new original approach, particularly regarding the influence of psychological factors, the need for gentle treatments, and the inappropriateness of bloodletting (**Figure 5**); thus breaking free from the contemporary dominant doctrine of his era.^14,15^

## EPILOGUE

Landré-Beauvais described RA to introduce a new arthritis type. Though his classification of RA as a relative of gout was inaccurate, his dissertation encouraged other researchers in the field of bone and joint disorders, like Garrod, to further study this disease. In our era, he is forgotten and unappreciated, overshadowed by the plethora of modern trends, still trying to establish his position in the history of rheumatology.
